# Supplementary material for: Localized inhibition in the Drosophila mushroom body
Source: eLife. 2020 Sep 21;9:e56954. doi: 10.7554/eLife.56954 (PMC7541083; doi:10.7554/eLife.56954)
Supplement: Supplementary file 1. [file elife-56954-supp1.docx]

Table S1: List of genotypes used

| Figure | Shorthand name / Purpose | Full genotype |
| --- | --- | --- |
| 1 | APL>GCaMP6f | 474-GAL4,mb247-dsRed/+; UAS-GCaMP6f/+ or  474-GAL4,mb247-dsRed/UAS-GCaMP6f; UAS-GCaMP6f/Sb |
| 1-supp1 | 474-GAL4>GFP | 474-GAL4/CyO; UAS-CD8::GFP |
| 2-supp1 | APL>Ort,GCaMP6f | NP2631,GH146-FLP/tub>Gal80>,UAS-Ort; UAS-GCaMP6f/UAS-Cherry |
| 3 | APL>GCaMP3, 853>dTRPA1 | UAS-dTRPA1/QUAS-GCaMP3; 853-GAL4/GH146-QF or UAS-dTRPA1/mb247-dsRed, QUAS-GCaMP3; 853-GAL4/GH146-QF |
| 3 | APL>GCaMP3, mb247>dTRPA1 | UAS-dTRPA1/QUAS-GCaMP3; mb247-GAL4/GH146-QF or UAS-dTRPA1/mb247-dsRed, QUAS-GCaMP3; mb247-GAL4/GH146-QF |
| 3 | APL>GCaMP3, c739>dTRPA1 | c739-GAL4/QUAS-GCaMP3; UAS-dTRPA1/GH146-QF or c739-GAL4/mb247-dsRed, QUAS-GCaMP3; UAS-dTRPA1/GH146-QF |
| 3 | APL>GCaMP3, NP3061>dTRPA1 | UAS-dTRPA1/mb247-dsRed, QUAS-GCaMP3; NP3061-GAL4/GH146-QF |
| 4 | APL>P2X2 | NP2631-GAL4,GH146-FLP,mb247-dsRed/tub>Gal80>, UAS-Cherry; UAS-P2X2/UAS-GCaMP6f |
| 4 | KC>P2X2, APL>GCaMP6f | 474-GAL4,mb247-dsRed/lexAop-P2X2; mb247-LexA/UAS-GCaMP6f(VK00005) |
| 4 | >P2X2 (negative control) | 474-GAL4,mb247-dsRed/UAS-GCaMP6f; +/lexAop-P2X2 |
| 6 | P2X2 (no driver, KC>GCaMP6f | UAS-P2X2(attP40)/+; MBLexA, LexAop-GCaMP6f/+ |
| 5-7 | APL>P2X2, GCaMP6f | UAS-P2X2(attP40)/mb247-dsRed; UAS-GCaMP6f/VT43924-Gal4.2-SV40 |
| 5-supp1 | VT43924-GAL4.2>GFP | VT43924-GAL4.2(attP2)/UAS-CD8::GFP |
| 6,7 | APL>P2X2, KC>GCaMP6f | UAS-P2X2(attP40)/+ ; mb247-LexA::VP16, lexAop-GCaMP6f/VT43924-Gal4.2-SV40 |
| 7 | KC>GCaMP6f | UAS-GCaMP6f/+ ; +/+ ; OK107-Gal4/+ |
| 7-supp1 | P2X2 (no driver, KC>GCaMP6f | UAS-P2X2(attP40)/+; MBLexA, LexAop-GCaMP6f/+ |
| 7-supp2 | KC>GCaMP6f | UAS-GCaMP6f/+ ; +/+ ; OK107-Gal4/+ |
